# High incidence of pulmonary tuberculosis in an indigenous Saharia tribe in Madhya Pradesh, central India—A prospective cohort study

**DOI:** 10.1371/journal.pgph.0000039

**Published:** 2022-06-10

**Authors:** Jyothi Bhat, Rajiv Yadav, Ravendra K. Sharma, Malaisamy Muniyandi, V. G. Rao

**Affiliations:** 1 ICMR–National Institute of Research in Tribal Health, Jabalpur, India; 2 ICMR- National Institute of Traditional Medicine, Belgavi, India; 3 Ch Charan Singh University, Meerut (U.P.), India; 4 ICMR–National Institute for Research in Tuberculosis, Chennai, India; University of California Irvine, UNITED STATES

## Abstract

**Background:**

Lack of TB incidence data is an important evidence gap among Saharia–a high TB burden indigenous community in Madhya Pradesh, central India. The present study was undertaken to calculate the incidence of pulmonary tuberculosis in this tribe.

**Objective:**

To calculate the incidence of pulmonary tuberculosis (PTB) among the Saharia tribal population in Madhya Pradesh, central India.

**Methods:**

A prospective cohort study was conducted among the Saharia tribal population (aged 15 years and above) of Shivpuri district in Madhya Pradesh state in central India. A total of 9,756 individuals were screened for TB in the baseline TB prevalence survey during 2012–13. All available household members were screened for symptoms suggestive of pulmonary TB (presumptive TB). Two sputum specimens were collected from persons having symptoms suggestive of TB and examined by smear and culture tests. A cohort of all non-TB individuals in the baseline survey was followed-up for one year and re-screened for the sysmptoms of PTB in the year 2014–15. Based on the data collected, the incidence of TB over one year period was estimated per 100,000 population.

**Results:**

A total of 9756 and 9044 individuals (≥ 15 years) were screened for symptoms suggestive of PTB during baseline and endline surveys respectively. The sputum specimens of presumptive TB cases were collected and examined by smear and culture tests. The overall incidence of bacteriologically positive (smear and/or culture) PTB over one year period was 1504 (95% Confidence Interval (CI): 1273–1776) per 100,000 in the study population, the incidence of smear-positive PTB was 1106 (95% CI: 910–1343), and the incidence of culture-positive PTB was 1084 (95% CI: 890–1319) per 100,000 population. The incidence for both smear and culture-positive PTB was 686 (95% CI: 535–878) per 100,000 population in the year 2014–15. The incidence of PTB was 2.8 times higher in males compared to females (2259 *vs* 807 per 100,000) and was positively correlated with age.

**Conclusion:**

The findings of the study, the first of its kind in the Saharia tribal population, indicate a high incidence of TB in this tribal community thereby highlighting the urgent need for focused and intensified efforts to achieve the goal of TB elimination in the country.

## Introduction

Millennium Development Goals in 2000 was a landmark step taken by 189 countries in their commitment to ‘meet the needs of the world’poorest. Goal number eight was to ‘stop the increase and start reversing the incidence of tuberculosis (TB) by 2015’[1]. Further, the Sustainable Development Goal (SDG) -3 reiterated reductionof TB incidence by 80% and TB deaths by 90% by 2030. To achieve this goal, information on the incidence of TB over a period is needed. The incidence studies are rarely conducted due to logistical problems and are very expensive. Hence, TB prevalence surveys and TB notifications are mainly used to estimate the incidence rates. Of the estimated 9.96 million incident cases of TB globally in 2019, 2.64 million occurred in India alone accounting for more than a quarter of the world’s TB cases [2].

In the year 2017, India set the target of ending TB in the country by the year 2025 and framed a New Strategic Plan (NSP). The NSP provides strategies to bring down the TB disease during the period 2015–2025 and aims at time -bound reduction of TB incidence from 217 per 100,000 in 2015 to 142, 77 and 44 per 100,000 in 2020, 2023 and 2025 respectively [3]. The NSP proposed bold strategies with adequate resources for the rapid decline of TB incidence by 2025 in the country. This is five years ahead of the global End TB targets under Sustainable Development Goalsto attain the vision of TB free India by 2025.

The high TB burden in the tribal population is a major challenge for the National TB Elimination Programme (NTEP) [4–6]. Limited access to health facilities combined with poverty, under-nutrition, tobacco and alcohol abuse makes management of TB difficult in tribal population. Saharia is one of the Particularly Vulnerable Tribal Groups (PVTGs) in the central Indian state of Madhya Pradesh and has the highest reported tuberculosis prevalence in the country ranging from 1270 to 3294 per 100000 population [7–9]. This is more than ten times compared to the national estimated prevalence of 320 per 100000 population. In contrast, the prevalence amongst two other PVTGs in the state, the Bharias and the Baigas, was found to be 430 and 150 respectively [5, 10]. Saharias usually inhabit a small group of hamlets known as ’Saharana’ adjacent to the main village. It is one of the most vulnerable populations suffering from poverty and unhygienic/ overcrowded living conditions. They are socio-economically underdeveloped with poor nutritional status. Most community members earn their livelihood as daily wage workers or labour in agriculture farms and migrate from one place to other in search of livelihoods [11, 12]. The remoteness of tribal habitats, low health literacy, practically non-existent public transport facilities, illiteracy and preference for traditional healers make TB management further difficult in Saharia tribal population [13–15]. Measuring, monitoring, and evaluating the performance of TB control activities by using available data is useful to formulate appropriate interventions and to modify TB control plans. Incidence of TB is one of the indicators of the risk of disease or the probability of developing the disease during the specified period. The present study was undertaken to calculate the incidence of pulmonary tuberculosis (PTB) among the Saharia tribe, a high TB burden community in Madhya Pradesh, central India.

## Methodology

### Study area and population

A prospective cohort study was conducted in thePohri block of Shivpuri district among Saharia tribal population. The total population of Pohari Block was enumerated as 207,352 in 2011 census. The highest concentration of the Saharia tribe is in the Pohari block with a population of 36,415 spread in128 villages [16]. The baseline TB prevalence survey was conducted in 53 selected villages in 2012–13 to cover the estimated sample sizeand to establish the cohort of non-TB population. All non-TB individuals in the baseline survey were followed-up for one year and re-screened for the symptoms of PTB in the year 2014–15.

### Data collection

A house to house census was carried out during the baseline survey by trained field investigators and all individuals in the household were registered. All available household members aged 15 years and above were screened for symptoms suggestive of TB such as persistent cough for ≥2 weeks, fever ≥1 month, chest pain for ≥1 month and history of haemoptysis within the last six months. Two sputum specimens were collected from the persons with any one of these symptoms (presumptive TB) and examined by smear and culture tests. The smear microscopy grading was done as per the NTEP guidelines. For culture, the samples were processed by modified Petroff’s method, inoculated on two slopes of Lowensten Jensen (LJ) medium and incubated for eight weeks. *M*.*tuberculosis* growth was confirmed by biochemical tests like niacin test, catalase test at 68°C and growth on LJ with PNB (500ug/ml).Non-tuberculous mycobacteria detected were also recorded and reported. Further, a house to house census was carried out in the endline survey and all non TB individuals in the baseline survey were revisited and screened for symptoms suggestive of TB. The same screening tool was used in both the baseline and endline surveys. All the detected cases were referred to NTEP for treatment. The project staff ensured that treatment was initiated.

### Sample size and sampling

The baseline TB prevalence survey was carried out in 2012–13 in the Saharia population residing in the Pohari block. The sample size was calculated as 9225 for the assumed prevalence of 1518 per 100,000 bacteriologically positive TB, with a precision of 25% at a 95% confidence level, a design effect of 2 for cluster sampling, and coverage for examination of at least 90%. A village was considered as a sampling unit for the study and all available eligible residents (15+ years) of selected villages were screened for symptoms of TB. The adjoining villages were selected to cover the estimated sample size of 9225 individuals. The cohort of individuals screened and found as non-TB in the baseline survey was followed up to one year for the occurrence of symptoms of TB. The endline survey was carried out in the year 2014–15 in the cohort of non TB individuals in the baseline survey. All the individuals included in the baseline survey were given an unique identification number for follow up in the endline survey.

### Data management

The data entry software was developed using the Census and Survey Processing System (CSPro) package version 5.0. The data entry format was developed with logical expressions and conditional statements were used to minimize the errors in data entry. Data were analysed using the Statistical Package for Social Sciences software, version 20.0 (IBM, Armonk, NY, USA). The incidence of pulmonary TB was calculated per 100,000 population with 95% confidence intervals and the incidence of PTB was further stratified by age and sex.

### Incidence of tuberculosis

A pulmonary TB case was defined as an individual whose sputum was positive for AFB by ZN microscopy and/or growth of *M*. *tuberculosis* by solid culture examination i.e. bacteriologically positive. Incidence of TB was defined as the number of new cases occurring within a period of one year time between baseline and endline surveys and was estimated per 100,000 population. Only bacteriologically confirmed cases from the endline survey were used to estimate the incidence. As per WHO, it is termed as “bacillary case incidence” between two surveys [7]. Since smear and culture are most commonly used diagnostic tools for TB diagnosis, seperate incidence estimates for smear and culture were provided along with the total incidence of tuberculosis.

### Ethical issues

The study was approved by the Institutional Ethics Committee of ICMR—National Institute of Research in Tribal Health, Jabalpur. The trained field investigators approached eligible individuals and explained the procedures, risks and benefits of the study in the local language. Written informed consent was obtained from all individuals willing to participate. In the case of minors, consent was obtained from the parents/guardians. Participation in the study was voluntary, and participants were able to withdraw at any time. All positive patients for TB were referred and linked to National Tuberculosis Elimination Programme (NTEP) and free counselling was provided to motivate patients to complete a full course of treatment.

## Results

**Fig 1:**
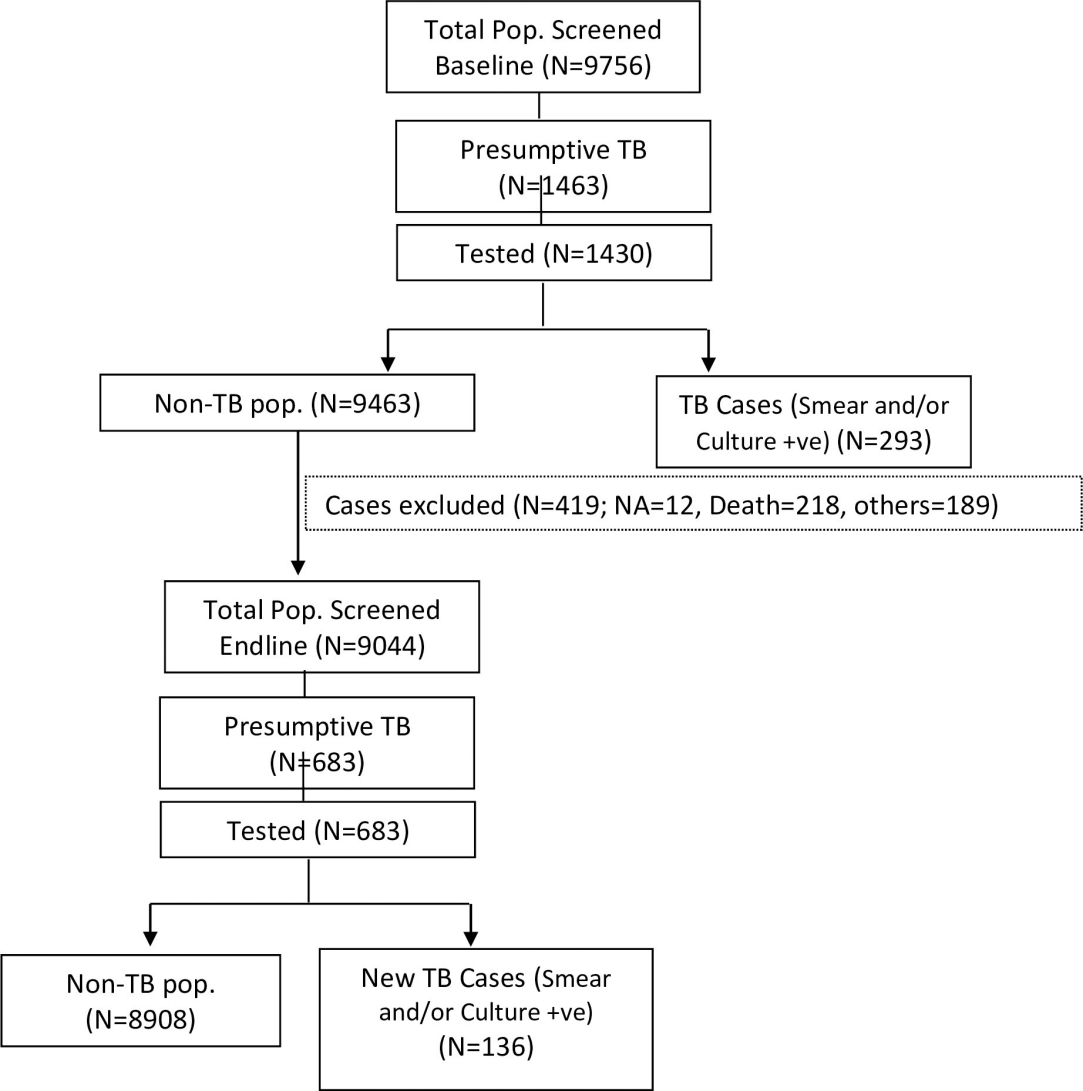
Flow chart of activities. Note: Detected NTB cases were excluded from TB cases.

A total of 9756 individuals were screened for the sysmptoms suggestive of PTB (Presumptive TB)_ at the baseline survey (Table 1 and Fig 1). Of these 1463 had symptoms suggestive of PTB and 293 were confirmed as bacteriologically positive PTB and were excluded. Among 9463 non-TB population enumerated in the baseline survey, 419 individuals were not included in the endline survey due to non-availability (12), death (218) and other reasons (189). (Table 2 & Fig 1). Thus, a cohort of 9044 non-TB individuals in the baseline survey was re-screened in the endline survey for sysmptoms of PTB. A total of 683 individuals had symptoms suggestive of PTB and 136 were bacteriologically positive.The calculated incidence of PTB in the Saharia tribe residing in the study area over the one year period was 1504 per 100,000 population (95% CI: 1273–1776). Of the 136 bacteriologically positive TB cases,100, 98 and 62 cases were diagnosed as smear- positive, culture- positive and both (smear and culture) positive new PTB cases respectively. The calculated incidence of PTB was 1106 (95% CI: 910–1343), 1084 (95% CI: 890–1319) and 686 (95% CI: 535–878) per 100,000 population for smear- positive, culture- positive and both (smear and culture) positive respectively

**Table 1:** Age-sex wise PTB cases (smear and/or culture positive) in the baseline survey.

Background characteristics		Popu Screened	Presumptive TB	Tested (Smear/culture)	PTB cases	Non TB population
Sex	Male	4781	1060	1038	231	4550
	Female	4975	403	392	62	4913
Age	15–24	2853	159	152	35	2818
	25–34	2616	335	325	66	2550
	35–44	1783	316	312	62	1721
	45–54	1391	355	346	78	1313
	55+	1113	298	295	52	1061
	Total	9756	1463	1430	293	9463

**Table 2:** Age-sex wise incidence of PTB (smear and/or culture positive) in the endline survey.

Background characteristics		Non-TB individuals	NA	Death	Others	Popu Screened	Presumptive TB	Tested	New TB cases	Incidence per 1,00,000	95% CI
Sex	M	4550	3	120	89	4338	475	475	98	2259	1857─2745
	F	4913	9	98	100	4706	208	208	38	807	589─1106
Age	15–24	2818	5	24	71	2718	69	69	17	625	391─999
	25–34	2550	3	15	52	2480	130	130	30	1209	848─1721
	35–44	1721	0	31	41	1649	163	163	37	2245	1633─3079
	45–54	1313	1	49	16	1247	182	182	29	2326	1624─3320
	55+	1061	3	99	9	950	139	139	23	2421	1619─3607
	**Total**	**9463**	**12**	**218**	**189**	**9044**	**683**	**683**	**136**	**1504**	**1273─1776**

**Fig 2:**
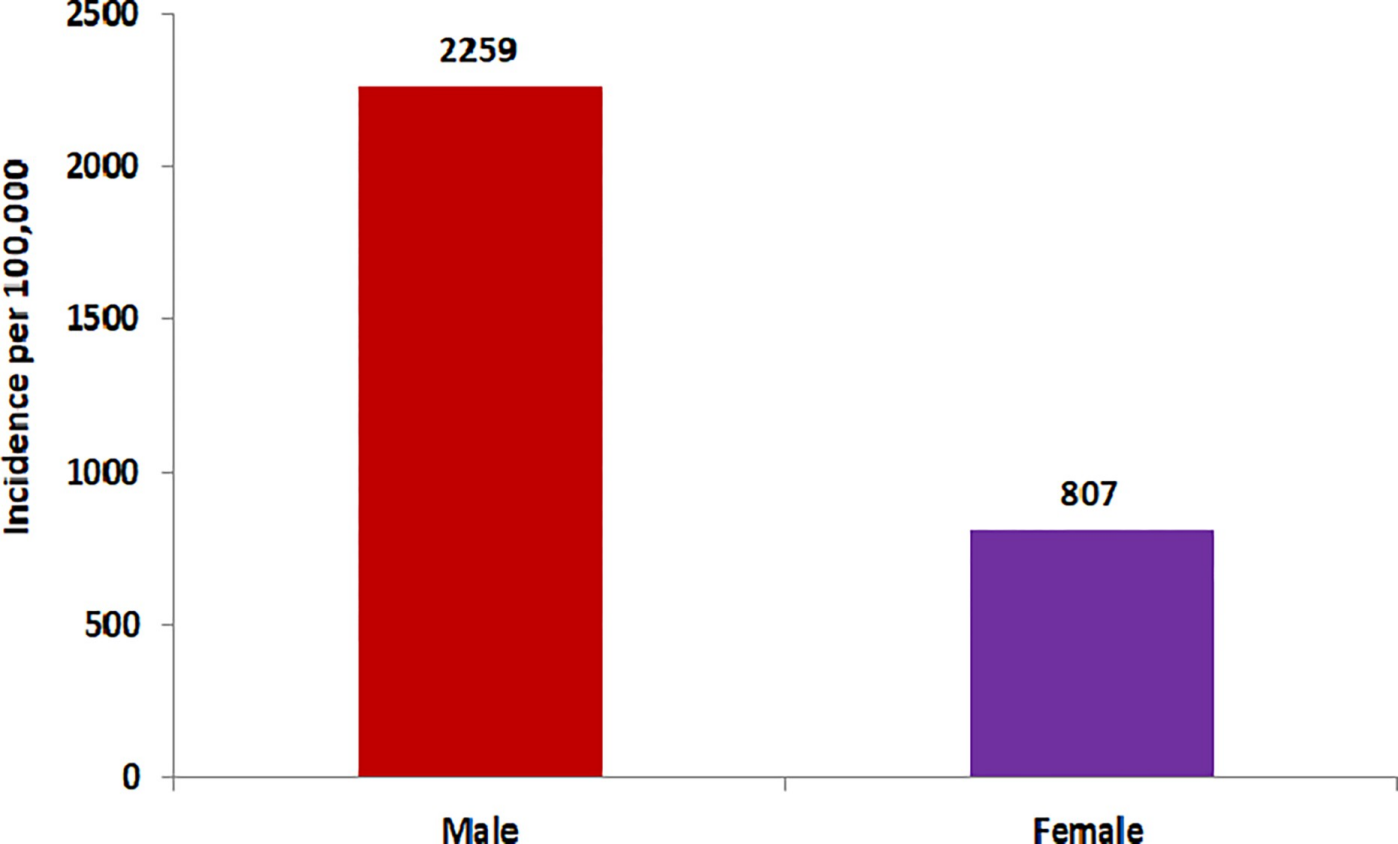
Incidence (smear and/or culture positive) of PTB by sex.

**Fig 3:**
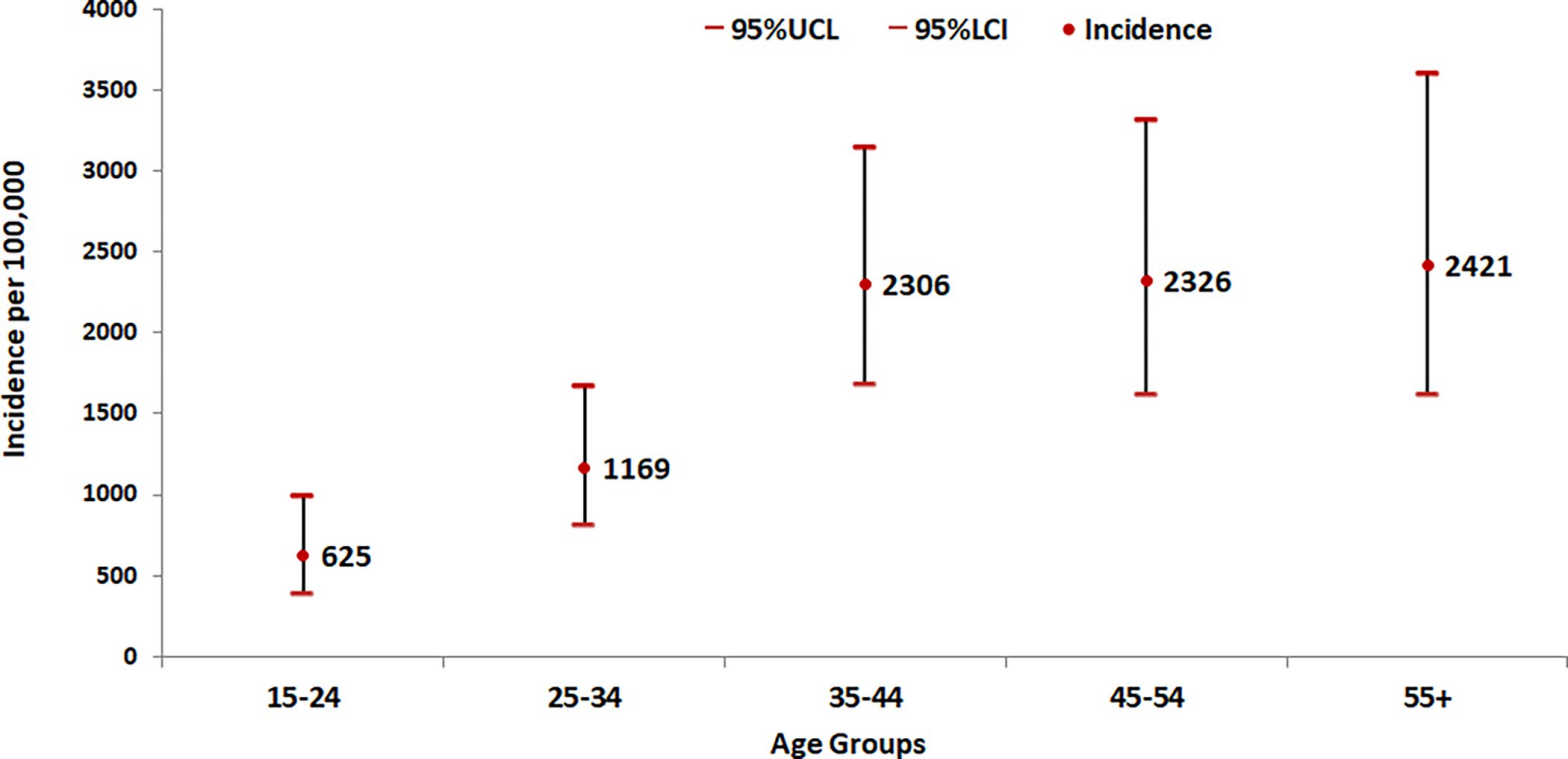
Agewise incidence (smear and/or culture positive) of PTB.

Concerning gender, the incidence of bacteriologically positive PTB was 2.8 times higher in males as compared to females (2259 *vs* 807 per 100,000) (Fig 2). The incidence rate was positively correlated with age, as incidence of bacteriologically positive (smear and/or culture) PTB increased with age. The highest incidence rate was noted among those aged 55 years and above (2421 per 100,000 population) (Fig 3).

The incidence of smear and culture positive cases was higher among males compared to females. The incidence of smear positive PTB was 3.1 times (1706 *vs* 552) and the culture positive PTB was 2.8 times higher (1637 *vs* 574 per 100000) among males as compared to females. It was also observed that as age increased, the incidence of smear and culture positive TB also increased. However, the highest incidence of smear-positive (1820 per 100,000 population) PTB was among 35–44 years, whereas culture positive PTB was highest among those aged 55 years and above (1895 per 100,000 population).

The incidence of both smear and culture-positive PTB was 3.4 times higher in males as compared to females (1083 *vs* 319 per 100000). It was also observed that as age increased, the incidence of both smear and culture-positive PTB also increased. The highest incidence of both smear and culture-positive PTB was among those aged 45–54 years and above (1123 per 100,000 population), which declined to 842 per 100000 (95% CI: 427–1653) among those 55 years and older ages. (Table 3)

**Table 3:** Age-sex and category wise incidence of PTB.

Category		Incidence per1,00,000	95% CI
**Smear-positive**			
Sex	Male	1706	1361─2136
	Female	552	377─808
Age	15–24	478	280─817
	25–34	967	651─1435
	35–44	1820	1278─2587
	45–54	1604	1041─2464
	55+	1368	801─2327
	**Total**	**1106**	**910─1343**
**Culture-positive**			
Sex	Male	1637	1300─2059
	Female	574	395─833
Age	15–24	478	280─817
	25–34	766	491─1193
	35–44	1517	1030─2230
	45–54	1844	1232─2752
	55+	1895	1202─2975
	**Total**	**1084**	**890─1319**
**Smear and Culture-positive**			
Sex	Male	1083	816─1438
	Female	319	193─525
Age	15–24	331	174─628
	25–34	564	336─945
	35–44	1032	645─1645
	45–54	1123	670─1876
	55+	842	427─1653
	**Total**	**686**	**535─878**

## Discussion

The present study is the first of its kind from India which was conducted among Saharia, one of the Particularly Vulnerable Tribal Groups (PVTG) in Madhya Pradesh, central India to estimate the incidence of PTB. Though TB incidence studies have been conducted in different settings [17–20], our findings have high validity since we have followed-up the same population at two time points to find out new PTB cases occurring during one year period. The salient finding from this study is the PTB incidence of 1504 per 100,000 population in Saharia tribe which is very high as compared to the national average of 217 per 100,000 population [3]. Similar findings of high TB burden have been reported among indigenous population globally [21, 22]. In India, studies conducted in other indigenous groups reported TB prevalence rates ranging from 133 to 840 per 100 000 in different parts of the country [5, 11, 23–26]. Our finding highlights an alarmingly high level of bacteriologically confirmed PTB incidence in this disadvantaged community. This information becomes very vital for policy makers and the NTEP being the baseline for monitoring the impact of various interventions for TB control in this population. The high TB incidence necessitates a relook at the current interventions being implemented among Saharias to control TB from an effectiveness perspective. During the study period, various interventions were offered to all the individuals in the cohort *viz*. door-to-door screening for tuberculosis and ensuring free DOT (Directly Observed Treatment) under NTEP. In addition, contact tracing and preventive tuberculosis treatment for children was also done in the study area as per NTEP guidelines.

On subgroup analysis, we found that the TB incidence was very high among males. Our findings corroborate with other studies reporting higher prevalence and incidence among male as compared to female [4–6, 8]. Similarly incidence of PTB was higher among elderly population. The current interventions need to be re-strategized to focus on these two sub populations which remain more vulnerable for disease acquisition. The peculiar issues faced by Saharia men in addition to higher TB incidence include high prevalence of tobacco smoking, alcohol consumption and migration to other areas for work [15, 27] The specific intervention strategies are therefore required to be devised and implemented to target this sub group such as enhancing smoking and alcohol cessation activities, local employment generation programmes etc. In addition, periodic health check-ups including TB detection and treatment at the village or nearer to their residence employing camp approach, mobile vans, culturally appropriate awareness programme might be useful intervention.

Our findings gain much importance in the present national scenario as Government of India is rigorously aiming and working for TB elimination in the country by the year 2025. The high TB incidence in Saharia tribe however, is a matter of concern and highlights the need for special efforts in order to achieve the goal of elimination. Under NTEP, the Tribal action plan provides for special incentives to the staff working in tribal areas. In addition, various stakeholders including the National and State Government, Development Partners, Civil Society Organizations, International Agencies, Research Institutions, Private Sector, and many others need to be involved for the cause of TB elimination in this high TB burden tribe and other trbal areas in the country. Though the performance of NTEP in tribal dominated districts is comparable to non tribal areas [28], we need to be vigilant considering wide variation in TB rates in different indigenous groups and varying exposure to the risk factors in different parts of the country. While we strive to achieve the goal of ‘TB elimination’at the national level, there is an urgent need to prioritise and accelerate exclusive research and to implement innovative and socially acceptable interventions to bring down the TB burden in this community at least nearer to the national average.

### Limitation of the study

In the present study, the findings are based on symptom screening alone. Due to non-availability of mobile X-ray unit, Chest X-ray could not done. This might have resulted in under-estimation of the true incidence of PTB in this indigenous community. In spite of this, the findings highlight high incidence of PTB in this tribe which may be useful in monitoring TB situation in this population.

Sputum was examined by smear and culture tests in the present study. Though non tuberculous mycobacteria could be a problem in some areas, it does not seem to a problem in Saharia tribe as our experience shows that proportion of NTM is less than 2%. Moreover, NTM on cultures were noted and informed to clinician.As care was taken to identify NTM, it is less likely that NTM is labelled as positive.

## Conclusion

The current evidence highlights very high burden of TB in terms of incidence in Saharia tribal community. This high TB burden warrants urgent need for focused intensified efforts for TB control and follow-up studies to monitor and evaluate the effectiveness of TB control measures particularly in this population in order to achieve the national goal of TB elimination.

## Supporting information

S1 Checklist STROBE statement—checklist of items for the study entitled “high incidence of pulmonary tuberculosis in an indigenous Saharia tribe in Madhya Pradesh, central India–a prospective cohort study”. (DOCX)

## References

[1] World Health Organization.Millenium Development Goals for tuberculosis. Geneva: World Health Organization 2007. http://www.who.int/tb/country/mdgs_for_tb/en

[2] World Health Organization. Global Tuberculosis Report 2020. World Health Organization, 2020. https://apps.who.int/iris/bitstream/handle/10665/336069/9789240013131-eng.pdf

[3] Central TB Division. National Strategic Plan for Tuberculosis Elimination 2017–2025. Central TB Division, Directorate General of Health Services, Ministry of Health with Family Welfare, Nirman Bhavan, New Delhi 2017.https://tbcindia.gov.in/WriteReadData/NSPDraft2020.02.2017201.pdf

[4] Rao VG, Muniyandi M, Bhat J, Yadav R, Sharma R. Research on tuberculosis in tribal areas in India: A systematic review. Indian J Tuberc 2018; 65(1): 8–14. doi: 10.1016/j.ijtb.2017.06.001 29332655

[5] Rao VG, Bhat J, Yadav R, Gopi PG, Selvakumar N, Wares DF. Prevalence of pulmonary tuberculosis among the Bharia, a primitive tribe of Madhya Pradesh, central India. Int J Tuberc Lung Dis 2010;14:368–70. 20132630

[6] Thomas Beena E., Adinarayanan Srividya, Manogaran C, Swaminathan Soumya. Pulmonary tuberculosis among tribals in India: A systematic review & meta-analysis. Indian J Med Res 2015; 141(5): 614–623. doi: 10.4103/0971-5916.159545 26139779 PMC4510760

[7] Chakma T, Vinay Rao P, Pall S, Kaushal LS, Datta M, Tiwari RS. Survey of pulmonary tuberculosis in primitive tribe of Madhya Pradesh. Indian J Tuberc 1996; 43: 85–9.

[8] Rao VG, Gopi PG, Bhat J, Selvakumar N, Yadav R, Tiwari B, et al. Pulmonary tuberculosis: a public health problem amongst the Saharia, a primitive tribe of Madhya Pradesh, Central India. Int J Infect Dis 2010;14:e713–6. doi: 10.1016/j.ijid.2010.02.2243 20605504

[9] Rao VG, Bhat J, Yadav R, Muniyandi M, Sharma R, Bhondeley MK. Pulmonary tuberculosis—a health problem amongst Saharia tribe in Madhya Pradesh.Indian J Med Res. 2015 May;141(5):630–5. doi: 10.4103/0971-5916.159560 26139781 PMC4510762

[10] Yadav R, Rao VG, Bhat J, Gopi PG, Selvakumar N, Wares DF. Tuberculosis prevalence among Baiga primitive tribe of Madhya Pradesh. Ind J Tuberc 2010; 57(2): 114–16. 21114182

[11] Sati VP. Sahariya Tribe: Society, Culture, Economy and Habitation. Ann Nat Sci. 2015;1(1): 26–31.

[12] Rajak J. Nutritional and Socio-Economic Status of Saharia Tribes In Madhya Pradesh. Int J Hum Soc Sci. 2016; 6(1):79–85.

[13] Muniyandi M, Rao VG, Bhat J, Yadav R, Sharma RK, Bhondeley MK. Health literacy on tuberculosis amongst vulnerable segment of population: special reference to Saharia tribe in central India.Indian J Med Res. 2015 May;141(5):640–7. doi: 10.4103/0971-5916.159566 26139783 PMC4510764

[14] Rao VG, Bhat J, Yadav R, Sharma RK, Muniyandi M. A comparative study of the socio-economic risk factors for pulmonary tuberculosis in the Saharia tribe of Madhya Pradesh, India.Trans R Soc Trop Med Hyg. 2018 Jun 1;112(6):272–278. doi: 10.1093/trstmh/try052 29931208

[15] Rao VG, Muniyandi M, Sharma RK, Yadav R, Bhat J. Long-term survival of patients treated for tuberculosis: a population-based longitudinal study in a resource-poor setting. Trop Med Int Health. 2021 Sep;26(9):1110–1116. doi: 10.1111/tmi.13636 34109699

[16] Census of India. (2011). Office of the Registrar General and Census Operation, Ministry of Home Affairs, Government of India, New Delhi, India.

[17] Brown M, Miiro G, Nkurunziza P, Watera C, Quigley MA, Dunne DW et al. Schistosoma mansoni, nematode infections, and progression to active tuberculosis among HIV-1-infected Ugandans. The American journal of tropical medicine and hygiene 2006; 74: 819–825. 16687687

[18] Chatterjee S, Kolappan C, Subramani R, Gopi PG, Chandrasekaran V, Fay MP et al. Incidence of Active Pulmonary Tuberculosis in Patients with Coincident Filarial and/or Intestina Helminth Infections Followed Longitudinally in South India. PLoS ONE 2014; 9(4): e94603. doi: 10.1371/journal.pone.0094603 24728010 PMC3984271

[19] Singh J, Sankar MM, Kumar S, Gopinath K, Singh N, Mani K et al. Incidence and Prevalence of Tuberculosis among Household Contacts of Pulmonary Tuberculosis Patients in a Peri-Urban Population of South Delhi, India. PLoS ONE 2013; 8(7): e69730. doi: 10.1371/journal.pone.0069730 23922784 PMC3724886

[20] Subramani R, Gomathy S, Lakshmi M, Swaminathan S. Trend in the incidence of smear-positive tuberculosis in a district in South India after DOTS implementation. Int J Tuberc Lung Dis 2016; 20(8):1022–1026. doi: 10.5588/ijtld.15.1018 27393534

[21] Tollefson D, Bloss E, Fanning A, Redd JT, Barker K, McCray E. Burden of tuberculosis in indigenous peoples globally: a systematic review. Int J Tuberc Lung Dis. 2013 Sep;17(9):1139–50. Epub 2013 Jul 2. doi: 10.5588/ijtld.12.0385 . 23823137 PMC6057791

[22] Cormier Maxime, Schwartzman Kevin, Dieynaba S N’Diaye, Claire E Boone, Alexandre M dos Santos, Júlia Gaspar, et al. Proximate determinants of tuberculosis in Indigenous peoples worldwide: a systematic review. Lancet Glob Health 2019; 7: e68–80. doi: 10.1016/S2214-109X(18)30435-2 30554764

[23] Narang P, Tyagi NK, Mendiratta DK, Jajoo UN, Bharambhe MS, Nayar S. Prevalence of sputum positive pulmonary tuberculosis in tribal and non-tribal populations of the Ashti and Karanja tahsils in Wardha district, Maharashtra State, India. Int J Tuberc Lung Dis 1999; 3(6): 478–82. 10383059

[24] Bhat J, Rao V G, Gopi P G, Yadav R, Selvakumar N, Tiwari BK et al. Prevalence of pulmonary tuberculosis amongst the tribal population of Madhya Pradesh, central India. Int J Epidemiol 2009; 38: 1026–1032. doi: 10.1093/ije/dyp222 19509182

[25] Datta M, Radhamani M P, Sadacharam K, Selvaraj R, Rao DL, Rao RS et al. Survey for tuberculosis in a tribal population in North Arcot District. Int J Tuberc Lung Dis 2001; 5: 240–249. 11326823

[26] Thomas BE, Thiruvengadam K, Vedhachalam C, A S Rao VG, Vijayachari P et.al. Prevalence of pulmonary tuberculosis among the tribal populations in India.PLoS One. 2021 Jun 4;16(6):e0251519. eCollection 2021. doi: 10.1371/journal.pone.0251519 . 34086684 PMC8177518

[27] Rao VG, Gopi PG, Bhat J, Yadav R, Selvakumar N, Wares DF. Selected risk factors associated with pulmonary tuberculosis among Sahariatribe of Madhya Pradesh, central India. Eur J public health 2012; 22: 271–3. doi: 10.1093/eurpub/ckr009 21324957

[28] Rao VG, Bhat J, Yadav R, Gopi PG, Selvakumar N, Wares DF. No time to be complacent with the performance of tuberculosis control activities in tribal areas of India. Int J Tuberc Lung Dis. 2011 Sep;15(9):1276–7. doi: 10.5588/ijtld.11.0427 . 21943864

